# Outcomes of Interventional-MRI Versus Microelectrode Recording-Guided Subthalamic Deep Brain Stimulation

**DOI:** 10.3389/fneur.2018.00241

**Published:** 2018-04-11

**Authors:** Philip S. Lee, Gregory M. Weiner, Danielle Corson, Jessica Kappel, Yue-Fang Chang, Valerie R. Suski, Sarah B. Berman, Houman Homayoun, Amber D. Van Laar, Donald J. Crammond, R. Mark Richardson

**Affiliations:** ^1^Department of Neurological Surgery, University of Pittsburgh School of Medicine, Pittsburgh, PA, United States; ^2^Department of Neurology, University of Pittsburgh School of Medicine, Pittsburgh, PA, United States; ^3^University of Pittsburgh Brain Institute, University of Pittsburgh, Pittsburgh, PA, United States

**Keywords:** deep brain stimulation, subthalamic nucleus, Parkinson’s disease, interventional MRI, microelectrode recording

## Abstract

In deep brain stimulation (DBS) of the subthalamic nucleus (STN) for Parkinson’s disease (PD), there is debate concerning the use of neuroimaging alone to confirm correct anatomic placement of the DBS lead into the STN, versus the use of microelectrode recording (MER) to confirm functional placement. We performed a retrospective study of a contemporaneous cohort of 45 consecutive patients who underwent either interventional-MRI (iMRI) or MER-guided DBS lead implantation. We compared radial lead error, motor and sensory side effect, and clinical benefit programming thresholds, and pre- and post-operative unified PD rating scale scores, and levodopa equivalent dosages. MER-guided surgery was associated with greater radial error compared to the intended target. In general, side effect thresholds during initial programming were slightly lower in the MER group, but clinical benefit thresholds were similar. No significant difference in the reduction of clinical symptoms or medication dosage was observed. In summary, iMRI lead implantation occurred with greater anatomic accuracy, in locations demonstrated to be the appropriate functional region of the STN, based on the observation of similar programming side effect and benefit thresholds obtained with MER. The production of equivalent clinical outcomes suggests that surgeon and patient preference can be used to guide the decision of whether to recommend iMRI or MER-guided DBS lead implantation to appropriate patients with PD.

## Introduction

Deep brain stimulation (DBS) for Parkinson’s disease (PD) was modernized over two decades ago (1–3) and has become the most common surgical treatment for this disorder. DBS lead implantation traditionally uses a combination of postmortem atlas-based coordinates and patient imaging for planning lead trajectories, with final trajectory refinement using microelectrode recordings (MER) to functionally map the target (4–6). However, the traditional MER approach can cause anxiety and discomfort for patients, since it performed awake to optimize recordings and test for efficacy and side effect thresholds with macrostimulation. Thus, anatomic verification approaches performed under general anesthesia without MER have been developed for DBS lead placement.

Anatomic approaches use imaging alone to verify lead placement. Stimulation in the sensorimotor region of the subthalamic nucleus (STN) is predictive of a good clinical motor effect for PD patients (7). Imaging lead locations after MER has shown that the sensorimotor region lies in the dorsolateral STN (8, 9). Thus, the functional maps obtained with MER have allowed the derivation of precise anatomic correlates (10) that can be used to predict successful lead placement without MER.

Initially controversial, the use of anatomic verification is increasing, with data indicating that implantation using intraoperative CT (11, 12) and intraoperative MRI for verification (13, 14) result in outcomes similar to those obtained with MER guidance. However, these techniques only verify lead location after placement. In contrast, interventional MRI (iMRI) relies on prospective stereotaxy, the use of real-time imaging to refine a trajectory that achieves target alignment prior to lead implantation (15). Using iMRI, DBS leads can be placed accurately (10, 16) and produce reductions in motor symptoms and medication dosage (17, 18) that are similar to MER (19, 20).

Despite these advances, some argue for the superiority of MER to anatomic approaches (21, 22). Central to this perspective is that MER often provides information that directs lead implantation away from the planned target (23). This may produce a discrepancy between the final targets for MER and iMRI approaches. Indeed, variability in lead location within the STN influences the clinical effect (7), and MER-directed adjustments may restore the lead closer toward the actual target by accounting for brain shift that can occur after cerebrospinal fluid (CSF) loss. When suboptimal lead placement occurs, programming changes can improve the clinical response and minimize untoward side effects. Thus, programming differences may reflect subtle lead location variability within the sensorimotor STN. So far, there is no direct comparison of stimulation thresholds between MER and iMRI.

We compared the effects of awake STN DBS lead placement guided by MER to asleep placement with iMRI. We compared lead placement error and postoperative change in UPDRS score and levodopa equivalent dosage (LED) between these approaches. Finally, we compared the side effect and clinical benefit thresholds at initial programming between these approaches. Our study, therefore, asked a somewhat different question from other recent, related studies, which is whether there is a functional difference in the response to intitial programming between these two patient groups. This is an important question, given that other studies typically have failed to acknowledge that accuracy related to an image-based target may not be the most relevant feature when compared with a technique that is designed to functionally map an area irrespective of the imaging findings.

## Materials and Methods

We performed a retrospective review of consecutive patients who underwent bilateral STN DBS for medically refractory PD between August 2012 and December 2014. The Institutional Review Board of the University of Pittsburgh approved this study, and all patients signed informed consent to be included in a research database. Patients either had leads placed while awake under local anesthesia with MER, or asleep under general anesthesia for iMRI-guided placement. Patients were referred by a multidisciplinary movement disorders team, based on each patient’s on–off medication evaluation, symptom complex, and lack of medical or surgical contraindication. Patients underwent iMRI placement according to their preference or judgment of the team that the patient would be a better candidate for an asleep procedure (e.g., severe symptoms incompatible with an awake procedure while off medication, general level of disability, or significant anxiety concerning an awake procedure). All surgeries were performed by a single surgeon (RMR).

### Operative Technique

Microelectrode recording patients underwent a high-resolution CT scan after Leksell stereotactic frame placement (Elekta, Stockholm, Sweden). Brainlab iPlan software (Brainlab, Feldkirchen, Germany) was used to fuse the CT scan with a preoperative 3 T MRI and plan initial stereotactic coordinates. Initial targeting was based on stereotactic coordinates for the STN of: *x* = ±12, *y* = −3, and *z* = −4, relative to the midpoint between the anterior and posterior commissures. The final trajectories were refined using direct targeting of the STN on a T2-weighted axial MRI sequence. The presumed center of the sensorimotor STN was targeted, at least 2.5 mm from the medial border, aligned in the anterior–posterior plane with the anterior border of the red nucleus. A single microelectrode was placed; if mapping was unsatisfactory, additional tracts were placed 2 mm anteriorly, posteriorly, medialy, or laterally. The top and bottom of the STN was identified using MER. The lead was placed at the bottom of the STN in the tract having an adequate span of presumed STN activity and “kinesthetic” single units exhibiting firing rate responses to limb movements. Intraoperative stimulation ensured the absence of unwanted side effects and evaluated clinical efficacy.

**Figure 1:**
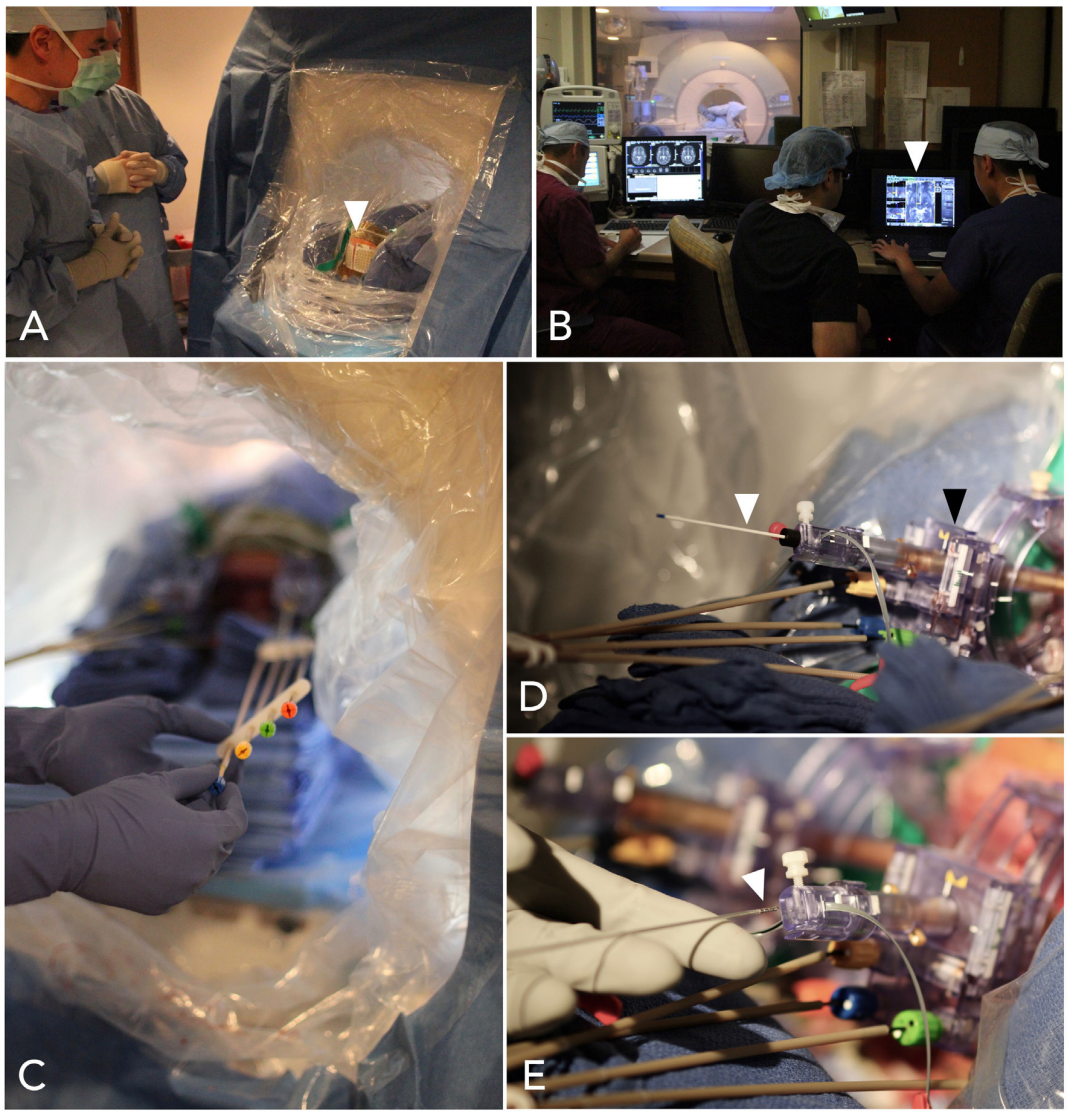
Steps in interventional-MRI-guided deep brain stimulation (DBS) lead implantation. **(A)** The patient is positioned supine and the head is sterilely draped within the MRI bore. MRI-visible marking grids (white arrowhead) are affixed to the scalp. **(B)** Volumetric scans are obtained and transferred to the ClearPoint planning station (white arrowhead), operated by the surgeon near the MRI console. **(C)** Instructions from the software are used to align the trajectories, by turning knobs affixed to a skull-mounted guide-frame. **(D)** When the guide-frame (black arrowhead) has been aligned such that the trajectory has a predicted radial error less than 0.5 mm, a ceramic stylet (white arrowhead) is placed to target depth, through a thin plastic sheath, and a scan is obtained to confirm accurate location. **(E)** A DBS lead (white arrowhead) is then placed through the sheath to target depth.

All iMRI procedures were performed on a 1.5 T Siemens Magnetom (Siemens AG, Munich, Germany). Stereotactic localization and navigation was performed using the Clearpoint NeuroNavigation system (MRI interventions, Irvine, CA, USA); technical details of using this system have been described elsewhere; and are briefly summarized in Figure 1 (24). Initial targeting was the same as for MER. The scalp was opened, the Clearpoint SmartFrame was mounted, and bur holes were drilled without opening the dura. MRI sequences were then acquired for targeting, using visible anatomic landmarks. The Clearpoint software calculated the error between the planned trajectory and the alignment of gadolinium-filled fiducials on the frame cannula; a cutoff of <0.5 mm was used as the criteria for proper frame alignment. After frame alignment, the dura was pierced with a sharp ceramic stylet and the lead was placed.

### Lead Error

Lead placement error was defined as the radial deviation between the planned target on the pre-implantation MRI and the center of the lead artifact in the plane 4 mm below the AC-PC line on the post-implantation MRI. Post-implantation MRIs were obtained for the MER patients on postoperative day 1 and at the conclusion of the procedure for iMRI patients. For iMRI, two raters measured radial deviation and the agreed error was recorded. For MER patients, pre- and post-operative MRIs were fused using the iPlan software. For some patients, the MER results guided lead placement into a different tract. In those instances, the error was measured from the mapping-based corrected trajectory. Two experienced raters independently measured the radial deviation on the iPlan software.

### Initial Programming

Initial DBS programming was performed by an experienced movement disorders neurologist or physician assistant at least 4 weeks after implantation. Standard test stimulation for threshold analysis was performed in monopolar mode with the case positive and each lead systematically tested. Stimulation benefit was divided into: rigidity, bradykinesia, tremor, and gait. Side effect profiles were: motor, sensory, or associational. Clinical benefit was tested at each lead contact in monopolar stimulation, with a stepwise voltage increase by tenths of a volt from 0 V. The contact was assigned as the negative dipole and the case as positive. Clinical benefit was noted if there was a subjective symptom improvement at a given voltage. A side effect (transient or permanent) was noted if it persisted for more than 10 s for sensory or association effects in order to reduce false positives; motor side effects (i.e., muscle contraction) were noted if they occurred immediately after increasing the voltage. The minimum voltage needed to produce a benefit or side effect was recorded.

### Outcome Measures

Pre- and postoperative UPDRS scores and LED were obtained for each patient. Preoperative data were obtained at the pre-surgical appointment. Postoperative data were obtained at the appointment closest to 6 months post-surgery. LED was calculated according to established guidelines (25).

### Statistical Analyses

Independent-sample *t*-tests were performed to evaluate group differences in lead error, UPDRS scores, and LED. Pearson correlation was used to compute lead measurement inter-rater reliability. Programming data were log transformed prior to analyses, due to skewness. Analysis of covariance (ANCOVA) was performed to control for differences in lead error, while evaluating group differences in percent change in UPDRS scores and LED, and programming thresholds. For the ANCOVAs, group (iMRI, MER) was the fixed factor with lead error as covariates. A *p*-value of ≤0.05 was considered statistically significant. Standard statistical processing software was used (SPSS version 22, IBM, Armonk, NY, USA).

## Results

### Patient Demographics

Sixty-five PD patients underwent DBS implantation in the study period. Nineteen patients were excluded due to either selection of a non-STN target (i.e., globus pallidus pars interna) or unilateral placement. One patient undergoing iMRI was excluded due to abortion of the procedure after detection of a small cortical hemorrhage (asymptomatic on postoperative exam). Thus, 45 patients were included; 21 iMRI and 24 MER. Post-operative follow-up data was not available for three patients in the iMRI group and three patients in the MER group, due to their return to neurology practices outside of our hospital system. Outside medical records received for these subjects did not include adequate descriptions of the outcome measures, but were sufficient for determining that all subjects were alive and receiving DBS therapy. There was no difference in age between the iMRI (M = 64.9 years, SD = 9.9 years) or MER (M = 66.3 years, SD = 6.6 years) groups, *t* (43) = −0.53, *p* = 0.60. In addition, there was no difference in time to post-operative follow-up between the iMRI (M = 7.7 mos, SD = 4.2 mos) or MER (M = 9.2 mos, SD = 6.2 mos) groups, *t* (37) = −0.93, *p* = 0.36. Pre-operative UPDRS scores and LED are listed in Table 1. There was no group difference in pre-operative on-medication UPDRS scores, *t* (40) = 1.71, *p* = 0.10, or daily levodopa equivalent dose, *t* (36) = −1.08, *p* = 0.29. However, there was a trend toward greater pre-operative off-medication symptom severity in the iMRI group, *t* (40) = 1.93, *p* = 0.06. Of note, no lead revisions occurred in either group.

**Table 1:** Means, SDs, and significance level of pre- and post-operative UPDRS scores and levodopa dosing equivalents.

	Interventional MRI	Microelectrode recording	*p*-Value
**Pre-operative UPDRS^a^**			0.10
On medication	24.8 (11.5)	19.5 (8.1)	0.06
Off medication	48.9 (14.3)	40.3 (14.6)	0.92
On versus off medication percent change	48% (21%)	49% (16%)	
**Post-operative UPDRS, on medication^a^**			
On stimulation			
On stimulation versus	19.6 (8.9)	16.3 (7.5)	0.16
pre-operative off medication percent change	59% (18%)	58% (14%)	0.15
On stimulation versus			
pre-operative on medication percent change	21% (22%)	12% (33%)	0.31
**Levodopa dosing equivalents^b^**			
Pre-operative	1,060.4 (523.6)	1,254.9 (577.0)	0.28
Post-operative	671.9 (476.9)	659.1 (411.3)	0.93
Pre- versus post-operative percent change	35% (38%)	43% (31%)	0.60

*^a^Total raw score*.

*^b^In milligrams*.

### Lead Placement

**Figure 2:**
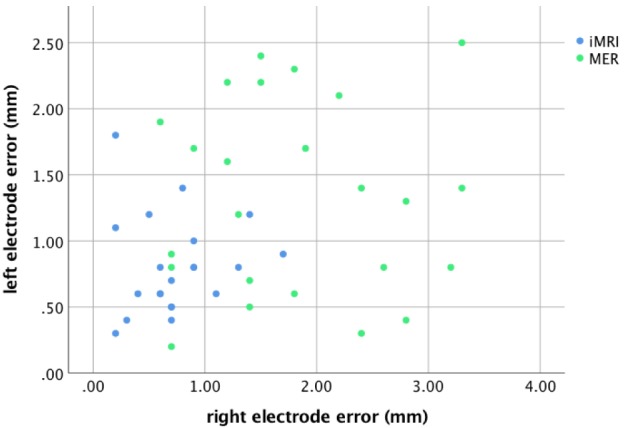
Scatterplot demonstrating subthalamic nucleus lead placement error for each subject.

For the MER group, there was a correlation between the rater’s measurements for both the left, *r* = 0.97, *p* < 0.001, and right leads, *r* = 0.98, *p* < 0.001; therefore, the mean rater measurement was used for comparisons. The radial lead placement error for each side is demonstrated in Figure 2. Four patients (17%) had lead placement in a tract other than center for the left side, while five patients (21%) had non-center placement on the right. Two of the four patients with non-center left lead placement were noted to have a mapping result that did not involve the identification of any kinesthetic cells, but had lead placement confirmed with satisfactory intraoperative stimulation (i.e., appropriate thresholds for clinical benefit and side effects). For the right side, all five patients with non-center placement and five others with center track placement had a non-kinesthetic mapping result, but satisfactory intraoperative stimulation. The lead error for the subset of patients with non-kinesthetic mapping (M = 1.8 mm, SD = 1.0 mm) did not differ from those in which mapping identified kinesthetic cells (M = 1.4 mm, SD = 0.8 mm), *t* (46) = −1.51, *p* = 0.15. These two groups of patients, therefore, were combined for all subsequent analyses. All iMRI patients had leads placed in the planned trajectory.

The lead anatomical radial errors in the axial target plane are listed in Table 2. The MER group lead error was greater than the iMRI group for both the left, *t* (43) = −2.96, *p* = 0.005, and the right side, *t* (43) = −5.44, *p* < 0.001. There was no significant difference between the sides, *t* (44) = −1.87, *p* = 0.08; therefore, in subsequent outcome analyses independent of sidedness (i.e., UPDRS and LED), the mean of the left and right side error for each patient was used.

**Table 2:** Means, SDs, and significance level of subthalamic nucleus (STN) lead to placement error in the axial targeting plane, in millimeters.

	Interventional MRI	Microelectrode recording	*p*-value
Left STN	0.8 (0.4)	1.3 (0.7)	0.005
Right STN	0.7 (0.4)	1.8 (0.9)	<0.001

### Side Effect Thresholds During Initial Programming

Analysis of covariances were performed to control for the effect of lead error on programming thresholds. Thresholds for the right and left side were combined for group comparisons. Side effect and benefit thresholds are listed in Table 3. Overall, there were lower side effect thresholds for the MER group. The MER group had a lower threshold for side effects at testing of contact 0, *F*(1,63) = 8.34, *p* = 0.005, contact 2, *F*(1,56) = 7.82, *p* = 0.007, and contact 3, *F*(1,56) = 4.14, *p* = 0.05, and a trend toward a lower threshold at contact 1, *F*(1,56) = 3.67, *p* = 0.06.

**Table 3:** Means, SDs, and significance level for voltage at which any side effect or clinical benefit was attained.

	Interventional MRI		Microelectrode recording		
			
	Mean	SD	Mean	SD	*p*-Value
**Side effects**					
Contact 0	2.6	0.9	1.7	1.1	0.003
Contact 1	2.8	1.0	2.3	1.1	0.06
Contact 2	3.0	0.9	2.1	1.1	0.007
Contact 3	2.8	0.9	2.1	1.3	0.05
**Clinical benefit**					
Contact 0	1.5	0.9	1.4	0.6	0.38
Contact 1	1.6	0.7	1.4	0.7	0.004
Contact 2	1.9	0.9	1.5	0.7	0.08
Contact 3	1.7	0.9	1.4	0.8	0.48

Overall there were slightly lower clinical benefit thresholds in the MER group. This was significant at contact 1, *F*(1,69) = 8.99, *p* = 0.004, but not at contact 0, *F*(1,67) = 0.80, *p* = 0.38, contact 2, *F*(1,56) = 3.24, *p* = 0.08, or contact 3, *F*(1,56) = 0.52, *p* = 0.48.

### Clinical Outcome

Post-operative UPDRS scores and LED are also listed in Table 1. ANCOVAs were performed to examine post-operative outcome with lead placement error as covariates. There was no difference in percent change of UPDRS scores in response to stimulation while on medication, *F*(1,35) = 1.04, *p* = 0.32, or in percent change of LED, *F*(1,38) = 0.28, *p* = 0.60.

### Complications

In the MER group, there were five complications: one deep vein thrombosis, one postoperative atrial fibrillation, one right facial nerve palsy, and one postoperative infection. The right facial nerve palsy presented 24 h after surgery with no infarct on imaging, was diagnosed as a Bell’s palsy and resolved. The infection occurred 2 months post-operatively at a bur hole incision, and a MRI demonstrated a brain abscess. The system was removed and the patient was treated with 6 weeks of intravenous antibiotics. The system was replaced after a full recovery by the patient. In the iMRI group, there were three complications: postoperative status epilepticus, unilateral lead fracture, and postoperative infection. The patient in status epilepticus was treated with anti-epileptic medication and required intubation for airway management until seizures were controlled; the patient subsequently made a full recovery. No etiology was found to account for the seizure onset. The lead fracture occurred at the lead extender and was replaced. The infection was a stitch abscess at a bur hole incision 2 months post-operatively. The patient fully recovered after surgical debridement without hardware explanation and 6 weeks of intravenous antibiotics.

## Discussion

We compared lead placement error, clinical outcome, and programming thresholds between patients undergoing DBS lead placement for PD, guided by either MER or iMRI. We found greater lead placement error in the MER group. Overall, the MER group exhibited lower side effect thresholds during initial programming, but similar clinical benefit thresholds, compared to the iMRI group. Despite these differences, we found equivalent clinical outcomes between the two groups, as measured by change in UPDRS scores and LED. Therefore, our data suggest that iMRI may be more anatomically accurate than MER, with comparable functional and clinical outcomes.

There has been considerable debate over the relative risks and benefits of functional versus anatomic verification of DBS lead placement (12, 22, 26–31). Unfortunately, one cannot control for differences in targeting with MER that reflect functional differences within the STN rather than anatomic differences visible on imaging. Moreover, drawing conclusions concerning targeting from examinations of outcome while on stimulation ignores the fact that programming settings are modified to account for variability in lead placement and to optimize the clinical effect. Therefore, we attempted to incorporate all of this variability by comparing the approaches on multiple levels. This study is the first to our knowledge that compares both the initial and long-term stimulation effects of DBS lead placement in the STN, between MER and iMRI approaches, in a contemporaneous cohort. Our findings are consistent with research that demonstrates similar accuracy and clinical outcome between MER-based strategies (4, 23) and non-MER direct targeting approaches (11, 13, 14, 18, 32, 33). Thus, our results add further confirmation that anatomic validation of targeting is equivalent to traditional functional validation with MER.

Our results confirm that iMRI is highly accurate for anatomic verification of the placement of DBS leads. One potential source of error that is not typically accounted for in this and other studies was the error inherent in the registration of the preoperative MRI and stereotactic CT used for planning, which is generally felt to be quite small (34). However, a possibly greater source of lead placement error is brain shift that occurs after the opening of dura and loss of CSF, which can occur in both traditional awake procedures with MER (35, 36) and in iMRI (37). In our study, we found that our mean error of iMRI was sub-millimetric (i.e., 0.8 mm, SD = 0.4 mm on the left, and 0.7 mm, SD = 0.4 mm on the right), compared to a mean error of 1.3 mm (SD = 0.8 mm) on the left and 1.8 mm (SD = 0.8 mm) on the right using MER. One possible reason for this discrepancy is that using iMRI, all leads were placed with a single penetration, thereby limiting CSF loss and brain shift. Furthermore, the increased error in our MER group for the right lead suggests that greater brain shift occurs over the course of the surgical case, as the left lead was always placed first. Our iMRI accuracy was similar to other iMRI studies (10, 38, 39), and was superior to results for CT-based verification approaches, which report mean errors of approximately 1.2 mm (SD = 0.7 mm) (11, 12). The greater accuracy of iMRI is likely a product of prospective stereotaxy, as unlike CT-based approaches, the trajectory can be adjusted prior to placement to account for brain shift.

The greater accuracy of iMRI for DBS lead placement may have beneficial clinical consequences. There was a higher side effect stimulation threshold during initial programming for iMRI versus MER. This suggests that iMRI leads were located further away from tracts surrounding the STN that can be inadvertently stimulated. Combined with roughly equivalent clinical effect thresholds, this indicates that iMRI results in functionally accurate placement of leads. In addition, iMRI potentially reduces the number and effect of hemorrhagic complications, since only one tract is necessary for lead placement. In contrast, MER and CT verification cannot detect a placement error until after placement. Thus, subsequent tracts may be needed for final implantation, which has a greater likelihood of hemorrhagic complications (40). Moreover, iMRI allows for the immediate detection of hemorrhages, which can then be rapidly addressed. Indeed, one iMRI patient was excluded because his surgery was aborted for this reason. This complication was asymptomatic, which highlights the likelihood that it would have been undetected during a traditional surgery. Finally, using general anesthesia for iMRI allows for greater patient comfort, making DBS available to patients whose anxiety or severe symptoms have prevented them from pursuing an awake procedure.

One assumption of anatomic targeting is uniformity of the optimum stimulation target within the STN across patients. This assumption is based on findings demonstrating that the sensorimotor region is located in the dorsolateral STN (7, 10, 41). However, there are age-dependent anatomic changes of the STN over time and functional STN subregions are not fully defined (42). The strength of MER is the ability to account for this variability through functional mapping. Thus, our greater lead error using MER may be a product of individual functional differences. Nonetheless, clinical outcomes were similar between our two groups, despite lead accuracy differences. It may be that small differences in accuracy do not manifest on global outcome indices, like UPDRS scores and LED changes. Indeed, the STN is comprised of different sub-regions that project diffusely to multiple brain regions responsible for more than just sensorimotor function (43). Future research should examine the effects of these differences in accuracy on other outcome measures, such as neurocognitive or speech changes that may occur in some patients after DBS (44).

Other limitations of this work include the absence of blinding of the programmers and the fact that patients were not randomized. Patients specifically requested, or were recommended for, iMRI DBS placement. This selection bias was likely reflected by more severe off-medication symptomatology in the iMRI group. Although, this difference was not statistically apparent, the strong trends toward greater pre-operative off-medication symptom severity and older age in the iMRI group suggest that the iMRI patients were more symptomatic in the off condition at baseline. Post-operative off-medication UPDRS scores also were not available. Therefore, off-medication comparisons of on- and off-stimulation symptoms could not be made. We also lack longer term follow-up for these groups. Symptoms of PD worsen over time despite intervention (13). A longer follow-up interval would delineate whether changes in outcome and programming thresholds occur after chronic stimulation. Finally, we hypothesized that greater lead placement error in the MER group might be due to uncompensated brain shift at the time of the postoperative MRI, however, we did not measure brain shift on pre-, intra-, and post-operative imaging.

## Conclusion

There is debate as to whether to rely on indirect or direct targeting methods for optimal DBS lead placement in the STN for PD. We found that a direct targeting approach using iMRI resulted in lower lead placement error and higher side effect programming thresholds. Despite this difference, MER and iMRI were equivalent in short-term outcome. Therefore, both approaches can be used to accurately position DBS leads and the choice can be driven by patient and clinician preference.

## Ethics Statement

This study was carried out in accordance with the recommendations of the University of Pittsburgh Human Research Protections Office, with written informed consent from all subjects. All subjects gave written informed consent in accordance with the Declaration of Helsinki. The protocol was approved by the Institutional Review Board of the University of Pittsburgh.

## Author Contributions

PL, GW, DC, JK, VS, SB, HH, AV, DC, and RR collected the data. PL, GW, Y-FC, and RR analyzed the data. PL, GW, DC, and RR wrote the manuscript.

## Conflict of Interest Statement

RR is the recipient of funding from Medtronic for research unrelated to this study. The submitted work was carried out in the absence of any personal, professional, or financial relationships that could potentially be construed as a conflict of interest.
